# Effect of an Extremely Low-Frequency Electromagnetic Field on the Concentration of Salivary Immunoglobulin A

**DOI:** 10.3390/ijerph19105786

**Published:** 2022-05-10

**Authors:** Piotr Skomro, Danuta Lietz-Kijak, Olga Bogdziewicz-Wałęsa, Joanna Janiszewska-Olszowska

**Affiliations:** 1 Department of Propaedeutic, Physical Diagnostics and Dental Physiotherapy, Pomeranian Medical University in Szczecin, Al. Powstańców Wlkp. 72, 70-111 Szczecin, Poland; piotr.skomro@pum.edu.pl (P.S.); danuta.lietz.kijak@pum.edu.pl (D.L.-K.); niezabodka@wp.pl (O.B.-W.); 2 Department of Interdisciplinary Dentistry, Pomeranian Medical University in Szczecin, Al. Powstańców Wlkp. 72, 70-111 Szczecin, Poland

**Keywords:** Extremely Low-Frequency Electromagnetic Field, immunity, Viofor JPS device, saliva, IgA, parotid gland, probiotics, postbiotics, paraprobiotics

## Abstract

Extremely Low-Frequency Electromagnetic Field (ELF MF) therapy is effective in the treatment of injury, inflammation and postoperative complications. Its clinical applications relate to bone unification, pain reduction, soft tissue oedema and the decrease of electric potentials in the oral cavity. It enhances regeneration of periapical bone lesions. It is obvious that cells (leukocytes, platelets, keratinocytes, osteoblasts) and proteins (fibrin, collagen, elastin and growth factors) exhibit alterations when exposed to an Extremely Low-Frequency Electromagnetic Field. The aim of the study was to evaluate the effect of an Extremely Low-Frequency Electromagnetic Field (ELF MF) on the parotid gland on the concentration of salivary immunoglobulin A. The study group consisted of 24 patients, aged 14–16, who underwent ELF MF on the parotid gland region. The control group comprised 25 matching persons. The IgA concentration in saliva samples was established using radial immunodiffusion. Following ELF MF, a statistically significant increase in the concentration of secretory immunoglobulin A was found in the study group, whereas in the control group, no statistically significant differences were noted. It can be concluded that an Extremely Low-Frequency Electromagnetic Field increases the activity of the immune system of the parotid gland.

## 1. Introduction

Any physical stimulus may affect tissues, systems and the entire human organism, leading to reduced inflammation, improved circulation, enhanced immunity and decreased or increased excitability of nerves. The influence observed improves the effects of treatment as well as accelerates regeneration of affected tissues and their recovery to physiological efficiency. Moreover, the analgesic effects in the area under treatment, as well as the degenerative effects of pathological tissues, are incredibly significant [1].

Extremely Low-Frequency Electromagnetic Field therapy is effective in the treatment of injury, inflammation and postoperative complications [2,3]. Its most effective clinical applications relate to bone unification, pain reduction [4], soft tissue oedema [3] and a decrease of electric potentials in the oral cavity [5]. It enhances regeneration of periapical bone lesions [6,7]. It is obvious that cells (leukocytes, platelets, keratinocytes, osteoblasts) and proteins (fibrin, collagen, elastin and growth factors) exhibit alterations when exposed to an Extremely Low-Frequency Electromagnetic Field [2,7,8]. However, no influence of low frequency magnetic fields on the content of salivary minerals has been found [9].

Extremely low-frequency magnetic field (ELF-MF) therapy is based on the application of a weak, slow-alternating electromagnetic field. The frequency of the basic waveform ranges from a few to 3000 Hz. Magnetic flux density is 1 pT–15 μT [10]. The alternating electromagnetic field exerts an analgesic effect on the human body, by increasing the secretion of endogenous opiates from the β-endorphin group. This action can be attributed to the modulation of neuronal activity as well as the secretion of melatonin by the pineal gland [11]. Apart from the direct effect on the opioid system, the alternating electromagnetic field has an indirect anti-inflammatory effect. Another very important therapeutic effect that can be achieved with the use of the fields is the anti-swelling effect, which is especially appreciated in the treatment of post-surgical complications. The most noteworthy advantage of the alternating magnetic field in the therapeutic process is increasing blood flow in arterial vessels and capillaries, stimulating oxygen utilization and cellular respiration. Other significant aspects include its impact on wound healing and beneficial effects of tissue regeneration following mechanical or thermal damage and other states of interruption of tissue continuity [12,13]. In addition, it should be emphasized that the abovementioned analgesic, anti-inflammatory and regenerative effects are some of the main objectives pursued by the therapist in the treatment following dental and surgical procedures [14].

The salivary immunoglobulin A is a specific immune factor produced by plasma cells derived from B lymphocytes localized in the vicinity of secretory epithelia. It constitutes about 5–15% of total salivary proteins. In contrast to this in serum, salivary IgA does not function as an opsonizing agent, since normally, neither cytotoxic T cells nor the complement system are present in saliva. Thus, the main function of salivary IgA is the inhibition of bacterial adherence and colonization by blocking surface structures involved in binding [15].

Higher concentrations of salivary IgA have been found in dental caries-resistant patients. It has been found that chewing gum increases the salivary flow rate, but it decreases IgA concentration [16]. Prolonged exercising, on the other hand, causes a significant increase of salivary IgA concentration with an unchanged IgA secretion rate and a significantly decreased salivary flow rate [17,18]. Some taste stimuli have been shown to increase salivary IgA concentration stimulation, with, for example, capsaicin significantly increasing the rate of saliva secretion and the amount of IgA secreted per minute [19].

No studies have been found concerning the influence of a low-frequency magnetic field on the IgA concentration in saliva.

The aim of the present study was to assess if local Extremely Low-Frequency Electromagnetic Field therapy interventions used for the treatment of periodontal periapical lesions have any influence on the concentration of salivary immunoglobulin A.

## 2. Materials and Methods

**Figure 1:**
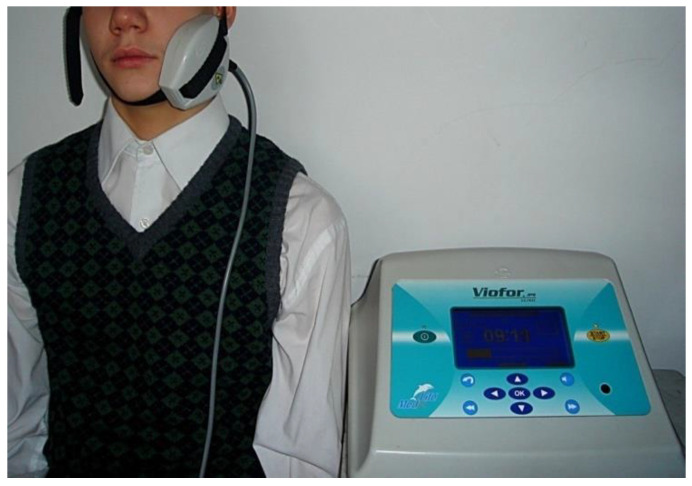
ELF EMF treatment with the use of an elliptical R applicator.

The study complied with ethical standards, and all participants and their guardians signed a written informed consent form and were informed about the technique and course of the research. Participants in the study did not receive any financial incentive and could withdraw from the study at any time. All the patients and their parents gave their written consent for saliva sampling and analysis. The principles outlined in the Declaration of Helsinki were carefully followed. The study included 49 patients aged 14–16 years referred for treatment of osteolytic periapical lesions of teeth. The same proper endodontic treatment was applied in both groups. The study group comprised 24 patients. The control group comprised 25 patients. In the study group, an Extremely Low-Frequency Electromagnetic Field was additionally used in the treatment. No Electromagnetic Field was applied in the control group. Study participants met the following inclusion criteria: general good health, lack of preexisting medical conditions, no bleeding, no generalized inflammation in the body and no medication that would affect their eligibility for treatment. We excluded patients on regular drug therapy and those with mental illness, coagulopathy, diabetes and chronic infections. None of the subjects were addicted to nicotine, alcohol or illegal drugs. Physiotherapy was carried out using a VIOFOR JPS device (Med & Life, Komorów, Poland). The magnetic fields of magnetostimulation generated Viofor JPS system have a pulsed character with a complex pulse shape and signal structure of signals giving a multi-spectrum frequencies. The fundamental frequencies of the waveform were in the range of 180–195 Hz. The pulse parcel frequencies were between 12.5 and 29 Hz, parcel groups were 2.8–7.6 Hz and series were 0.08–0.3 Hz. The magnetic field induction for the Viofor JPS device was 50–1200 μT. The amplitude of the current flowing through the applicator coils for the intensity level was 1.5–2.0 A. There is a choice of 3 methods, M1–3; three programs, P1–3, programmed by the manufacturer, and an intensity range from 0.5–12. In the study group, the M1 method, P3 program and intensity of 6 were selected from the Viofor JPS device. Treatments with an ELF EMF were each time provided with elliptical R applicators, used topically, generating a uniform field (Figure 1). 

Treatment was carried out once a day, for 10 min, five days a week (excluding Saturdays and Sundays) for 15 days. The elliptic R applicator utilizing an ELF EMF and ionic cyclotron resonance was set in the area of the parotid gland. Cyclotron resonance involves the repeated transfer of energy by an alternating electric field to particles circulating in a magnetic field that are only those whose frequency of circulation is the same as the frequency of the stimulating field. The applicator dimensions were 9.5 cm × 7.5 cm × 2 cm. The application surface was. approx. 20 cm^2^ (circle of approx. 5 cm diameter). The value of the magnetic field induction B [μT] for the R elliptical applicator at the intensity of 6 was 600 μT. 

The M1 method is recommended for the treatment of chronic conditions. Method M1 means that the intensity level remains constant for the entire duration of the program. Only the direction of the field polarization changes, factory set with the programmed time for this program. The application time was set to 10 min.

**Figure 2:**
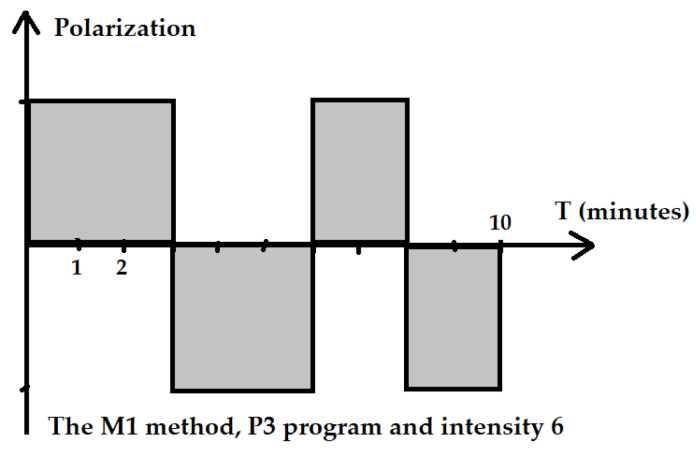
Scheme of the application of the ELF EMF of the M1 method and P3 program.

The P3 program is recommended by the manufacturer in case of the need to obtain quick effects or in a patient with a low sensitivity to the magnetic field. For the P3 program, the cycle of changes had the following course: 3 min of positive polarization, 3 min of negative polarization, 2 min of positive polarization and 2 min negative polarization of the electromagnetic field (Figure 2). For the R elliptical applicator in program P3 with an intensity of I = 6, the parameters were as follows: the average power in the pulse was 40.3 mW, the power density in the pulse was 10.7 mW/cm^2^, energy delivered during the treatment was 24.2 J and energy density was 1.23 J/cm^2^. 

Non-stimulated mixed saliva (6 mL) was sampled in a single-use plastic container once a week in every patient included in the study, in a fasting state or at least two hours after a solid or liquid meal, after prior mouth rinsing with water at room temperature. The sampling was conducted in a separate room and a comfortable sitting position. In the study group, saliva sampling was carried out before magnetic stimulation and after the 5th, 10th and 15th ELF EMF interventions. Patients from the control group had saliva sampled three times before routine visits for dental treatment.

The immunoglobulin A concentration was measured by means of radial immunodiffusion using BINDARID sets (The Binding Site, Birmingham, UK). 

Data normality was verified using a Shapiro-Wilk test. The Wilcoxon’s matched pairs rank and Student *t*-test were used to compare the data obtained. The significance level of *p* < 0.05 was used as reference for all comparisons.

## 3. Results

The IgA concentrations in the study group before thee Extremely Low-Frequency Electromagnetic Field therapy and after 5, 10 and 15 interventions are presented in Table 1.

A gradual increase in the content of salivary IgA was apparent. The values of the immunoglobulin A concentration (mg/mL) in the saliva of patients using metal prosthetic restorations before the treatments with the Viofor JPS system ranged from 62.5 to 159.0 mg/mL (me = 100.2 mg/mL); after 5 treatments, it ranged from from 85.0 to 159.8 mg/mL (me = 127.2 mg/mL); after 10 treatments, it ranged from 99.0 to 170.2 mg/mL (me = 137.4 mg/mL) and after 15 treatments, it ranged from 122.7 to 190.0 mg/mL (me = 156.1 mg/mL). Highly statistically significant differences (*p* < 0.001) were obtained for values before treatments and values after 10 and 15 treatments and between values after 5 and 15 treatments. Between the other three comparisons, the differences were highly statistically significant (*p* < 0.004). The IgA concentrations in the control group are presented in Table 2.

In these patients, no statistically significant changes were found in the immunoglobulin A concentration.

**Figure 3:**
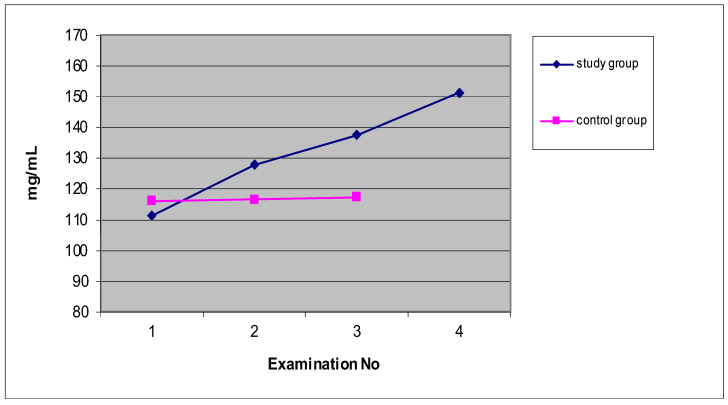
The mean values of the concentration of immunoglobulin A (mg/mL) in the saliva of patients from the study group (Table 1) and the control group (Table 2).

Figure 3 shows that the mean values of the concentration of immunoglobulin A (mg/mL) in the saliva of the patients of the study group were characterized by a statistically significant upward trend of 36.1% between the values before and after the 15th treatment. However, in the control group, the mean values of the concentration of immunoglobulin A slightly increased (0.9%) between the first and third examinations. A characteristic feature was that the mean values of the three control group examinations were very similar and slightly higher than the mean value of the first examination of the study group patients who underwent ELF MF treatment.

## 4. Discussion

A standard level of immunoglobulin A in saliva is in question, since the concentration varies in relation to the origin of saliva, method of collection and stimulation of secretion. In order to facilitate diagnoses, Chang et al. [20] determined the efficacy of commercial oral fluid collection devices for their ability to recover three human immunoglobulin isotypes: immunoglobulin A (IgA), IgG and IgM. The sandwich enzyme-linked immunosorbent assay was used to determine the antibody. For all isotypes tested, the amount of antibody recovered from the device was dependent on the initial seeding concentration. Collectively, these data suggest that the product used for specimen collection can affect the retrieval of antibodies and potentially confound patient diagnosis.

In the present study, a significant span between the maximum and minimum values, as well as between the upper and lower quartiles was noted before ELF EMF therapy, both in the study and in the control group, indicating great individual variability. Therefore, we used pretreatment values as references. In the present study, after 15 interventions, an increase in the concentration of immunoglobulin A and a narrowness of the range between the maximum and minimum values, as well as of the upper and lower quartiles, was found. It could be suggested that a maximum secretion and concentration of less individual variability might have been reached after 15 interventions in most subjects.

This may result in their increased transport across cell membranes, affecting tissues and their components, like collagen, dentin, keratin, and other proteins. The effects of electromagnetic fields have also been demonstrated in the spinal cord, adrenal cortex, sex hormones, DNA and inner layers of cell membranes [21,22]. Their action influences the permeability of biological barriers. Analgesic effects of the electromagnetic field have been demonstrated by many researchers. The stimulation of immunological processes has also been shown [23,24,25]. The secretory immunoglogulin A (IgA) system is the principal arm of the immune response that protects the upper respiratory and enteric tracts from reinfection by pathogenic organisms, to which a specific immune response has already been elicited. This IgA response involves the local production of IgA by plasma cells in mucosal sites followed by its transport onto mucosal surfaces or into glandular secretions, such as saliva, bile and tears, via the polymeric immunoglobulin receptor (pIgR) [26]. Thus, an impaired IgA response could increase the risk of developing respiratory or enteric infections. Increased levels of total IgA but reduced levels of anti-Actinomycetes actinomycetemcomitans in whole saliva have been found in patients with periodontitis [27]. A significantly lower concentration of salivary IgA has been found in passively smoking children [28]. In the present study, the mean concentration of immunoglobulin A in saliva in the study group before ELF MF therapy interventions was slightly lower than that of the control group, which might confirm the presence of an inflammatory process. Higher values of salivary IgA were found in patients with Sjögren’s syndrome, reflecting autoimmune inflammation of the salivary glands [29]. Therefore, it seems that ELF MF as a method of treatment should be contraindicated in patients with autoimmune inflammation diseases.

Oral infections are an important problem in dentistry. This is in addition to cariogenic bacteria resulting in dental caries [30,31]. In susceptible patients, periodontal tissues can be exposed by pathogenic bacteria leading to periodontitis [32]. Therefore, various methods have been sought to counteract bacterial colonization in the oral cavity [33]. Among the proposed supportive methods, probiotics, postbiotics and paraprobiotics are often recommended. Probiotics represent a breakthrough in the treatment of oral inflammation. Unlike antibiotics, they do not produce adverse effects [34,35]. So far, probiotics of the genus Lactobacillus, administered in the form of pastilles, have been mainly used as an adjunct to periodontal treatment in periodontitis. Currently, probiotic preparations are introduced in the form of toothpastes as well as chewing gums. In addition, toothpastes contain probiotics of the genus Bifidobacterium, which, to date, have still been poorly studied [36]. In vitro and in vivo studies have shown that some postbiotics and paraprobiotics exhibit bioactive properties such as anti-inflammatory, immunomodulatory, antiproliferative, antioxidant and antibacterial activities. Thus, they may be health-promoting agents in observed clinical trials in humans [37]. Probiotics provide major health benefits by modulating the gastrointestinal microbiome. However, there are limitations, such as viability control. This hinders their full potential use in the food and pharmaceutical industries. Therefore, live probiotic bacteria are gradually being replaced by non-viable paraprobiotics and/or probiotic-derived biomolecules, so-called postbiotics [38]. Despite the scientific evidence, their mechanisms of action remain to be elucidated. Further research in various fields should be continued to better understand the relationship between their use and oral clinical conditions. 

## 5. Conclusions

Extremely Low-Frequency Electromagnetic Field therapy increases the activity of the secretion of IgA imnunoglobulin from the parotid gland. Further studies involving a larger number of patients and a longer follow-up time are needed.

## Figures and Tables

**Table 1:** Characteristics of the concentration of immunoglobulin A [mg/mL] in saliva before and after Extremely Low-Frequency Electromagnetic Field therapy in the study group.

Distribution Characteristics	Before the Treatment	After 5 Treatments	After 10 Treatments	After 15 Treatments
n	24	24	24	24
min–max	62.5–159.0	85.0–159.8	99.0–170.2	122.7–190.0
Q1–Q3	85.6–142.1	108.3–150.6	121.9–156.2	135.5–160.6
me	100.2	127.2	137.4	156.1
M ± SD	111.2 ± 31.6	127.9 ± 23.8	137.6 ± 19.5	151.3 ± 17.4
W	0.911	0.921	0.951	0.939
*p*	<0.04	>0.06	>0.28	>0.16
Wilcoxon Test	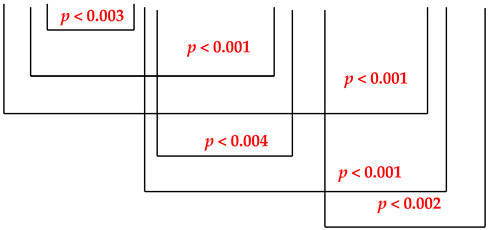			

n—group size, min—minimum value, max—maximum value, Q_1_—first quartile, Q_3_—third quartile, m_e_—median, M—arithmetic average, SD—standard deviation, W—Shapiro–Wilk Test, *p*–significance level.

**Table 2:** Characteristics of the concentration of immunoglobulin A [mg/mL] in saliva in the subsequent examinations of the control group.

Distribution Characteristics	1st Examination	2nd Examination	3rd Examination
n	25	25	25
min–max	72.5–170.2	67.8–165.3	70.3–160.8
Q1–Q3	97.7–129.3	97.8–130.8	97.4–130.5
me	120.3	115.3	120.3
M ± SD	116.2 ± 27.2	116.5 ± 29.0	117.3 ± 27.0
W	0.959	0.954	0.955
*p*	>0.41	>0.31	>0.33
Student’s *t*-test	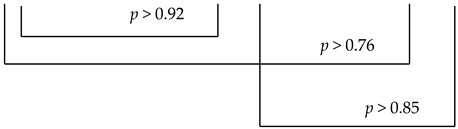		

n—minimum value, max—maximum value, Q_1_—first quartile, Q_3_—third quartile, m_e_—median, M—arithmetic average, SD—standard deviation, W—Shapiro–Wilk Test, *p*—significance level.

## Data Availability

Not applicable.

## References

[1] Pasek J., Pasek T., Sieroń- Stołtny K., Cieślar G., Sieroń A. (2016). Electromagnetic fields in medicine—The state of art. Electromagn. Biol. Med..

[2] Johnson M.T., Vanscoy-Cornett A., Vesper D.N., Swez J.A., Chamberlain J.K., Seaward M.B., Nindl G. (2001). Electromagnetic fields used clinically to improve bone healing also impact lymphocyte proliferation in vitro. Biomed. Sci. Instrum..

[3] Markov M. (2007). Magnetic field therapy. Electromagn. Biol. Med..

[4] Kopacz Ł., Ciosek Ż., Gronwald H., Skomro P., Ardan R., Lietz-Kijak D. (2020). Comparative Analysis of the Influence of Selected Physical Factors on the Level of Pain in the Course of Temporomandibular Joint Disorders. Pain Res. Manag..

[5] Skomro P., Lietz-Kijak D., Kijak E., Bohdziewicz-Wałęsa O., Opalko K. (2012). The change of electric potentials in the oral cavity after application of extremely low frequency pulsed magnetic field. Adv. Hyg. Exp. Med..

[6] Opalko K., Dojs A. (2006). Bone structure regeneration after low induction magnetic field treatment in teeth chosen for extraction. Adv. Med. Sci..

[7] Lietz-Kijak D., Kijak E., Śliwiński Z., Opalko K. (2013). The use of physiotherapy in the regeneration of periapical bone structures of the teeth, prepared to load the prosthetic. Adv. Hyg. Exp. Med..

[8] Takano-Yamamoto T., Kawakami M., Sakuda M. (1992). Effect of a pulsing electromagnetic field on demineralized bone-matrix induced bone formation in a bony defect in the premaxilla of rats. J. Dent. Res..

[9] Skomro P., Opalko K., Bohdziewicz O., Noceń I., Janiszewska-Olszowska J. (2009). Limited effect of low frequency magnetic fields on the concentrations of calcium, magnesium and fluoride in saliva. Magnes. Res..

[10] Okokon E., Roivainen P., Kheifets L., Mezei G., Juutilainen J. (2014). Indoor transformer stations and ELF magnetic field exposure: Use of transformer structural characteristics to improve exposure assessment. J. Exp. Sci. Environ. Epidemiol..

[11] Wilson B.W., Wright C.W., Morris J.E., Buschbom R.L., Brown D.P., Miller D.L., Sommers-Flannigan R., Anderson L.E. (1990). Evidence for an effect of ELF electromagnetic fields on human pineal gland function. J. Pineal Res..

[12] Pall M.L. (2013). Electromagnetic fields act via activation of voltage-gated calcium channels to produce beneficial or adverse effects. J. Cell Mol. Med..

[13] Leslie F. (1970). Distortion of twisted orientation patterns in liquid crystals by magnetic field. Mol. Cryst. Liq. Cryst..

[14] Santini M.T., Rainaldi G., Indovina P.L. (2009). Cellular effects of extremely low frequency (ELF) electromagnetic fields. Int. J. Radiat. Biol..

[15] Van Nieuw Amerongen A., Bolscher J.G.M., Veerman E.C.I. (2004). Salivary proteins: Protective and diagnostic value in cariology?. Caries Res..

[16] Van Anders S.M. (2009). Chewing gum has large effects on salivary testosterone, estradiol, and secretory immunoglobulin A assays in women and men. Psychoneuroendocrinology.

[17] Allgrove J.E., Geneen L., Latif S., Gleeson M. (2009). Influence of a fed or fasted state on the s-IgA response to prolonged cycling in active men and women. Int. J. Sport Nutr. Exerc. Metab..

[18] Davidson G., Allgrove J., Gleeson M. (2009). Salivary antimicrobial peptides (LL-37 and alpha-defensins HNP1-3), antimicrobial and IgA responses to prolonged exercise. Eur. J. Appl. Physiol..

[19] Kono Y., Kubota A., Taira M., Katsuyama N., Sugimoto K. (2018). Effects of oral stimulation with capsaicin on salivary secretion and neural activities in the autonomic system and the brain. J. Dent. Sci..

[20] Chang C.K., Cohen M.E., Bienek D.R. (2009). Efficiency of oral fluid collection devices in extracting antibodies. Oral Microbiol. Immunol..

[21] Wróbel M.P., Szymborska-Kajanek A., Wystrychowski G., Biniszkiewicz T., Sieroń-Słotny K., Sieroń A., Pierzchała K., Grzeszczak W., Strojek K. (2008). Impact of low frequency pulsed magnetic fields on pain intensity, quality of life and sleep disturbances in patients with painful diabetic polyneuropathy. Diabetes Metab..

[22] Robertson J.A., Juen N., Théberge JWeller J., Drost D.J., Prato F.S., Thomas A.W. (2010). Evidence for a dose-dependent effect of pulsed magnetic fields on pain processing. Neurosci. Lett..

[23] Robertson J.A., Thomas A.W., Bureau Y., Prato F.S. (2007). The influence of extremely low frequency magnetic fields of cytoprotection and repair. Bioelectromagnetics.

[24] Miyakoshi J. (2005). Effects of static magnetic fields at the cellular level. Prog. Biophys. Mol. Biol..

[25] Volpe P. (2003). Interactions of zero-frequency and oscillating magnetic fields with biostruc-tures and biosystems. Photohem. Photobiol. Sci..

[26] Mestecky J., Leu C., Russell M.W. (1991). Selective transport of IgA: Cellular and molecular aspects. Gastroenterol. Clin. N. Am..

[27] Bachrach G., Muster Z., Raz I., Chaushu G., Stabholz A., Nussbaum G., Gutner M., Chaushu S. (2008). Assessing the levels of immunoglobulins in the saliva of diabetic individuals with periodontitis using checkerboard immunodetection. Oral Dis..

[28] Auşar A., Darka Ö., Bodrumlu E.H., Bek Y. (2009). Evaluation of the relationship between passive smoking and salivary electrolytes, protein, secretory IgA, sialic acid and amylase in young children. Arch. Oral Biol..

[29] Helenius L.M., Meurman J.H., Helenius I., Kari K., Hietanen J., Suuronen R., Hallikainen D., Kautiainen H., Leirisalo-Repo M., Lindqvist C. (2005). Oral and salivary parameters in patients with rheumatic diseases. Acta Odont. Scand..

[30] Lutovac M., Popova O.V., Macanovic G., Kristina R., Lutovac B., Ketin S., Biocanin R. (2017). Testing the Effect of Aggressive Beverage on the Damage of Enamel Structure. Open Access Maced. J. Med. Sci..

[31] Colombo M., Poggio C., Lasagna A., Chiesa M., Scribante A. (2019). Vickers Micro-Hardness of New Restorative CAD/CAM Dental Materials: Evaluation and Comparison after Exposure to Acidic Drink. Materials.

[32] Deng Z.L., Szafrański S.P., Jarek M., Bhuju S., Wagner-Döbler I. (2017). Dysbiosis in chronic periodontitis: Key microbial players and interactions with the human host. Sci. Rep..

[33] Scribante A., Poggio C., Gallo S., Riva P., Cuocci A., Carbone M., Arciola C.R., Colombo M. (2020). In Vitro Re-Hardening of Bleached Enamel Using Mineralizing Pastes: Toward Preventing Bacterial Colonization. Materials.

[34] Ikram S., Hassan N., Raffat M.A., Mirza S., Akram Z. (2018). Systematic review and meta-analysis of double-blind, placebo- controlled, randomized clinical trials using probiotics in chronic periodontitis. J. Investig. Clin. Dent..

[35] Teame T., Wang A., Xie M., Zhang Z., Yang Y., Ding Q., Gao Ch Olsen R.E., Ran Ch Zhou Z. (2020). Paraprobiotics and Postbiotics of Probiotic *Lactobacilli*, Their Positive Effects on the Host and Action Mechanisms: A Review. Front. Nutr..

[36] Butera A., Gallo S., Molino C.D., Chiesa A., Preda C., Esposito F., Scribante A. (2021). Probiotic Alternative to Chlorhexidine in Periodontal Therapy: Evaluation of Clinical and Microbiological Parameters. Microorganisms.

[37] Cuevas-González P.F., Liceaga A.M., Aguilar-Toalá J.E. (2020). Postbiotics and paraprobiotics: From concepts to applications. Food Res. Int..

[38] Nataraj B.H., Ali S.A., Behare P.V., Yadav H. (2020). Postbiotics-parabiotics: The new horizons in microbial biotherapy and functional foods. Microb. Cell Fact..

